# Supplementary material for: Tunable Ampere phase plate for low dose imaging of biomolecular complexes
Source: Sci Rep. 2018 Apr 4;8:5592. doi: 10.1038/s41598-018-23100-3 (PMC5884816; doi:10.1038/s41598-018-23100-3)
Supplement: Supplementary file 1 — Supplementary Dataset 1 [file 41598_2018_23100_MOESM1_ESM.doc]

**Tunable Ampere phase plate for low dose imaging of biomolecular complexes**

**Amir H. Tavabi^1^, Marco Beleggia^2^, Vadim Migunov^1^, Alexey Savenko^3^, Ozan Öktem^4^, Rafal E. Dunin-Borkowski^1^, Giulio Pozzi^1, 5^**

1- Ernst Ruska-Centre for Microscopy and Spectroscopy with Electrons, and Peter Grünberg Institute, Forschungszentrum Jülich, 52428 Jülich, Germany

2- Center for Electron Nanoscopy, Technical University of Denmark, 2800 Kgs Lyngby, Denmark

3- FEI Company, Achtseweg Noord 5, 5600 KA, Eindhoven, The Netherlands

4- Centre for Industrial and Applied Mathematics, Department of Mathematics, KTH - Royal Institute of Technology, Stockholm, Sweden

5- Department of Physics and Astronomy, University of Bologna, Viale B. Pichat 6/2, 40127 Bologna, Italy

**Supplementary information**

**Phase plate fabrication**

A prototype tunable Ampere phase plate was fabricated as a free-standing three-dimensional nanoscale circuit using focused ion beam (FIB) milling. A chemically etched Au wire was sculpted in two orthogonal directions to form a Z-shaped device, in which the central segment (referred to as the “hook”) could be set parallel to the incident electron beam direction in the transmission electron microscope (TEM).

FIB milling was performed in an FEI Helios NanoLab 600i dual-beam system operated at 30 kV using Ga^+^ ions with beam currents in the range 7 pA-60 nA. In the final milling step, the desired 200 x 200 nm^2^ cross-section of the hook arm was achieved over a length of 2 µm. Another etched Au needle was milled in the form of a flat pad, in order to provide a reliable low-resistance contact to complete the electrical circuit.

The hook and needle were mounted in a NanoFactory scanning tunneling microscopy specimen holder (Fig. S1). By using the piezo-driven tip of the holder, contact was established between the end of the hook and the tip of the Au needle inside the TEM. The current-voltage characteristic of the device was measured to be linear (Fig. S2), confirming the formation of an Ohmic contact between the needle and the hook. The total resistance of the device was 22 Ω. A comparison between current-voltage (I-V) curves acquired with and without electron beam illumination revealed that the beam had no detectable effect on the resistance of the device.

**Off-axis electron holography**

Off-axis electron holography was used to study the phase shift associated with the magnetic field of the short segment of the Z-shaped phase plate, with closure of the electrical circuit provided by two horizontal wires connected to an external voltage supply. The magnetic field of the horizontal wires has a negligible effect on the phase shift of the incoming electrons.

Analysis of a holographic interference fringe pattern allows quantitative and non-invasive retrieval of the electron-optical phase shift


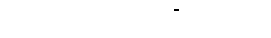
 (1)

after the electron beam has interacted with an object. In Eq. 1, *x* and *y* are directions in the plane of the specimen and *z* is the electron beam direction. The first term describes the contribution to the phase shift from the electrostatic potential within and around the object *V*(*x,y,z*), where *C_E_* is an interaction constant that takes a value of
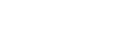
 at an accelerating voltage of 300 kV. The second term describes the magnetic contribution to the phase shift, where *A_z_*(*x*,*y*,*z*) is the *z* component of the magnetic vector potential and *e* and *ћ* are the absolute values of the electron charge and the reduced Planck constant, respectively.

A schematic diagram of the off-axis electron holography setup is shown in Fig. S3. Electrons emitted from a coherent field emission source illuminate the hook, which is displaced slightly from the optic axis of the microscope. After interacting with the hook, the electron wavefunction is magnified by the imaging lens (the Lorentz lens in our case) onto the first intermediate image plane, which is conjugate to the final recording plane *via* the magnifying lenses of the microscope. Before reaching the intermediate image plane, the electron wavefunction interacts with an electron biprism, which overlaps part of the electron beam that passed through the specimen (the object wave) with part that passed away from it (the reference wave). The resulting interference fringe pattern created in the first intermediate image plane (the hologram) captures information about the amplitude of the object wave and the phase difference between the object wave and the reference wave.

Off-axis electron holography was carried out in Lorentz mode (in magnetic-field-free conditions) using elliptical illumination in an FEI Titan 60-300 TEM operated at 300 kV. This microscope is equipped with an XFEG field emission electron source, an objective lens aberration corrector and two electron biprisms. The upper biprism, which is located between the aberration corrector and the diffraction lens of the microscope, was used to form a
2.2-*µ*m-wide interference region (Fig. S4). A biprism voltage of 107 V was used, resulting in a holographic interference fringe spacing of 2.8 nm. All phase images were reconstructed from electron holograms using home-written scripts.

The two-dimensional contour maps shown in Figs 1d - 1f display the cosine of 8 times the recorded phase shift. The superimposed colors, which represent the direction of the projected in-plane magnetic field, were generated from the gradient of the measured phase shift. The original phase images and linescans of the phase extracted from them are shown in Fig. S5.

**Theoretical model for the phase shift**

In order to calculate the phase shift due to the straight section of a current-carrying wire, we used the fifth formulation of Ampere's law:


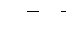
 . (2)

We considered a closed loop made from two wires of length *L* aligned parallel to the optical axis of the microscope, one at the origin and the other far away, say at (F,0,0), carrying opposite currents and connected by horizontal straight wires. According to Eq. 1 the contribution to the electron-optical phase shift from the horizontal straight wires is identically zero, while that due to the vertical wires is


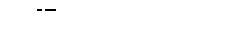
 . (3)

This expression results from integration along the optical axis of contributions to the phase shift from vertical current elements d*L*, where the vector potential of the element at the origin (0,0,0) is given by the expression


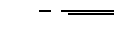
 . (4)

This equation for the *z* component of the vector potential is exactly analogous to that of the electrical potential of an elementary charge of magnitude *Q*= *iL*/*c*. The equivalent electrical charge for a 2-*µ*m-long wire carrying 1 mA of current is approximately 40 e.

If the reference wave is perturbed by the stray field of the wire, then, rather than retrieving the ideal object wavefunction


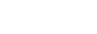
 , (5)

off-axis electron holography retrieves the phase shift of a fictitious object, whose wavefunction is given by the expression


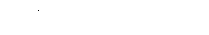
 , (6)

where *Θ* is the angle of the biprism with respect to the object in the chosen reference system, and *D* is the interference distance, which can be measured by recording two interferograms using different biprism potentials and measuring the variation in distance between recognizable features. The perturbed reference wave is an imaging artifact of the holography technique and, as discussed in the main text, does not affect the object being imaged, or the performance and functionality of the phase plate.

**Image formation with the TAPP**

During operation, the TAPP is placed in a diffraction plane of the microscope (the objective aperture plane or any of its conjugates further down the column) and modifies the phase relationship between scattered beams, thereby producing phase contrast.

Following standard image formation theory, the transfer function of the microscope in its simplest form is exp[i*χ*(**q**)], where the aberration function

*χ*(**q**)=*πλZq*^2^+*ϕ*(**q**) (7)

accounts for the TAPP phase shift *ϕ*(**q**) and a possible residual defocus (*Z*) term, while λ is the electron wavelength. If the effective camera length in the chosen diffraction plane is *L_c_* , then a lateral distance *x* from the wire corresponds to a spatial frequency of *q*= *x*/(λ*L_c_*) in the small-angle approximation sin(*θ*)≈*θ*.

Centering the vertical current segment of the TAPP at location (*q_c_*,0) in the Fourier plane, the phase shift from Eq. 3 is


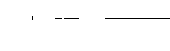
 , (8)

where an arbitrary phase offset has been included so that ϕ(0)=0.

Since the wire is opaque to electrons, a top-hat amplitude term is added to the transfer function, which becomes *A*(**q**)exp[i*χ*(**q**)], with *A*(**q**)=1 when |*q_x_-q_c_*|>*q_w_*/2 and zero otherwise. Here, *q_w_* = *w*/(λ*L_c_*) is the spatial frequency associated with a wire of width *w*.

For a wave object *ψ*(**r**)=*a*(**r**)exp[i*ϕ*_o_(**r**)], the image intensity at the detector is

*I*(**r**)=|FT^-1^{*s*(**q**)*A*(**q**)exp[i*χ*(**q**)]}|^2^ , (9)

where FT{} denotes a two-dimensional Fourier transform operation and *s*(**q**)=FT{*a*(**r**)exp[i*ϕ*_o_(**r**)]}.

**TAPP image simulations**

A nucleosome core particle (NCP) was chosen as a test object to assess the performance of the TAPP. The molecular structure of the NCP (PDB: 1AOI) [11], which is shown in Fig. 2a (as rendered using Chimera software), illustrates how the 146 DNA base pairs are assembled into a superhelix and surround a central histone protein octamer.

The amplitude *a*(**r**) and phase *ϕ*_o_(**r**) of the object *ψ*(**r**) corresponding to a single NCP embedded in a 50-nm-thick layer of ice were determined by propagating an initially-plane coherent electron wave through an electrostatic potential resulting from the superposition of atomic potentials. Inelastic scattering was taken into account by means of an absorption potential, as described in [12]. All inelastically scattered electrons were removed from the calculation, resulting in a zero-loss-filtered exit wave. The amplitude, which is shown in Fig. 2b, reveals that inelastic scattering from ice embedment “absorbs” about 40% of the incoming primary electrons, while additional elastic amplitude effects due to the NCP are limited to a marginal (2-3%) redistribution of electrons within the field of view. Visually, the amplitude image bears little resemblance to the NCP structure shown in Fig. 2a. In contrast, the phase shift in Fig. 2c shows a relatively large difference between electrons going through the ice support and those passing through the NCP and displays a remarkable resemblance to Fig. 2a.

As a benchmark, we first simulated the appearance of the NCP corresponding to Fresnel (out-of-focus) imaging. By setting *i* = 0 mA and *Z* = (0.05, 0.5, 1.0) μm in Eqs. 7 and 8, we calculated the images shown in Figs 2d-f, respectively. By setting, instead, *i* = (0.25, 0.6, 3.0) mA and *Z* = 0 µm in the same equations, we calculated the TAPP images shown in Figs 2g-i, respectively.

Other parameters that were used in the simulations were: accelerating voltage 200 kV (λ =2.5 pm); wire width *w* = 200 nm; vertical current segment length *L* = 2 μm; 1024 x 1024 pixels of size *r_0_* = 1 Å in real space and *q*_0_ = 9.7 μm^-1^ in Fourier space; camera length *L*_c_ = 8.25 mm, resulting in the wire being effectively 1 pixel wide
(*q*_w_ = *w*/(*λL*_c_) = 9.7 μm^-1^ = *q*_0_); vertical current segment centered at *q*= *q*_c_ = *q*_0_, such that the TAPP intercepts the row of pixels adjacent to that containing the direct beam at *q*= 0.

Figure S6 shows the NCP power spectrum |*s*(**q**)|, with 0.25 rad phase contours from a TAPP operated at 1 mA superimposed, and with annotations indicating relevant geometrical parameters. Both the wire width and its lateral displacement from the direct beam have been augmented for illustrative purposes.

Figure S7 illustrates the effects of the position and obstruction of the wire. The first column shows the optimal setup, where the camera length has been chosen to reduce the angle subtended by the 200 nm wire to 1 pixel, *i.e.*, *θ_w_* = *w*/*L*_c_ = λ*q*_0_ = 24.5 μrad, and the wire is positioned 1 pixel (*θ_c_* = *θ_w_*) to the right of *q*= 0. In this configuration, phase contrast (here “negative”, *i.e.*, produced by a current of the same magnitude but opposite to that considered in Fig. 2h) is high, in terms of both contrast and fidelity to the phase shift shown in Fig. 2c. Foucault effects, *i.e.*, the removal of some scattered intensity due to the presence of the opaque wire, are barely visible. Maintaining the same size of the wire while displacing it 10 pixels to the right (*θ_c_* = 10 *θ_w_*), as shown in the second column, further reduces obstruction effects, which are now essentially absent, but reduces the contrast since the net phase shift between the unscattered and scattered electrons is lower. The obstruction becomes relevant when the wire is effectively 10 pixels wide, which corresponds to a 2 μm wire at the same camera length, or, equivalently, to a 200 nm wire at an unrealistic camera length of 0.825 mm. This situaion is shown in the remaining columns, where the large wire (*θ_w_* = 245 μrad) is moved progressively away from *q*= 0, from 150 μrad (third column, with the edge of the wire adjacent to *q*= 0) to 392 μrad (fourth column) to 637 μrad (fifth column). Foucault effects are now visible, to the detriment of phase contrast. It should be noted that the use of a thick wire adjacent to the direct beam (third column) results in contrast that is higher than for the thin wire adjacent to the direct beam (first column). However, this is a mixed phase and amplitude effect, which is more of an artifact than an effect that could be exploited, since the fidelity is lower (with half of the object appearing brighter than the other half, which may lead to contrast misinterpretation).


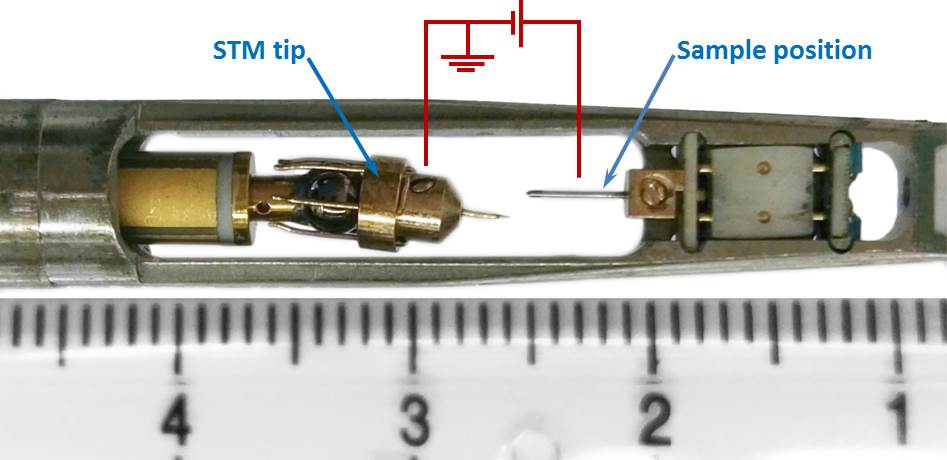


**Fig. S1** Photograph of the end of a NanoFactory scanning tunneling microscopy specimen holder, with a schematic representation of the electrical circuit used in the present experiments superimposed.


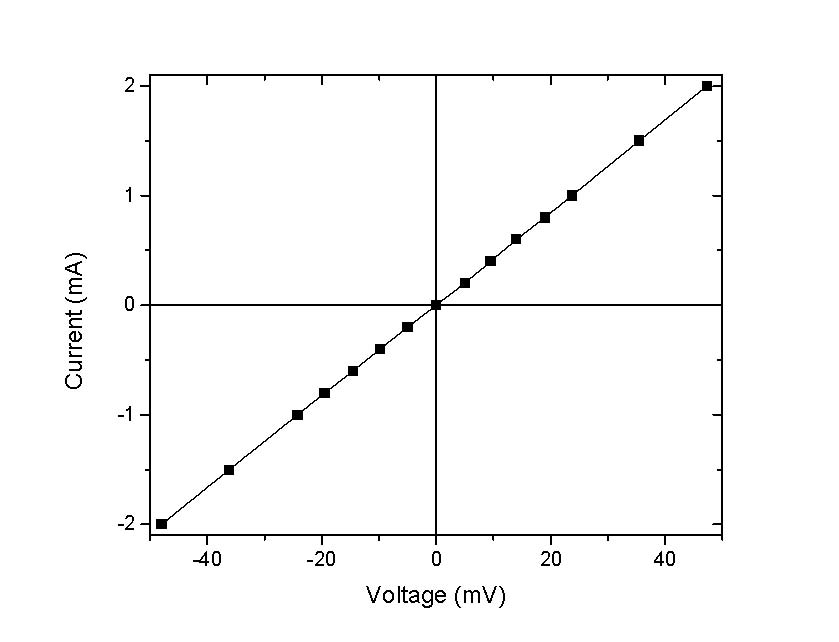


**Fig. S2** Current-voltage (*I*-*V*) characteristic of the TAPP device measured inside the transmission electron microscope illuminated by electron beam.


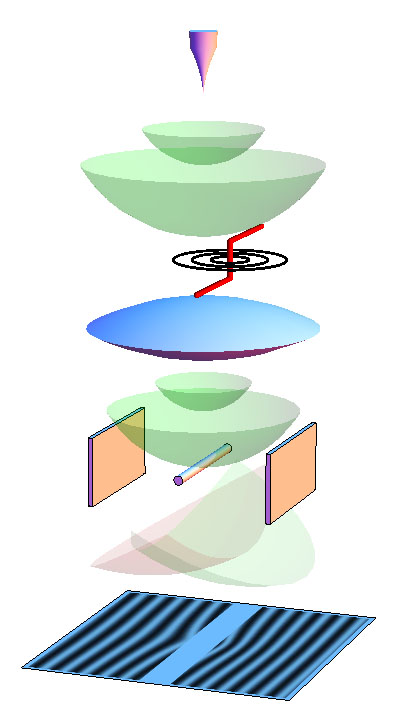


**Fig. S3** Schematic illustration of the off-axis electron holography setup in the TEM.


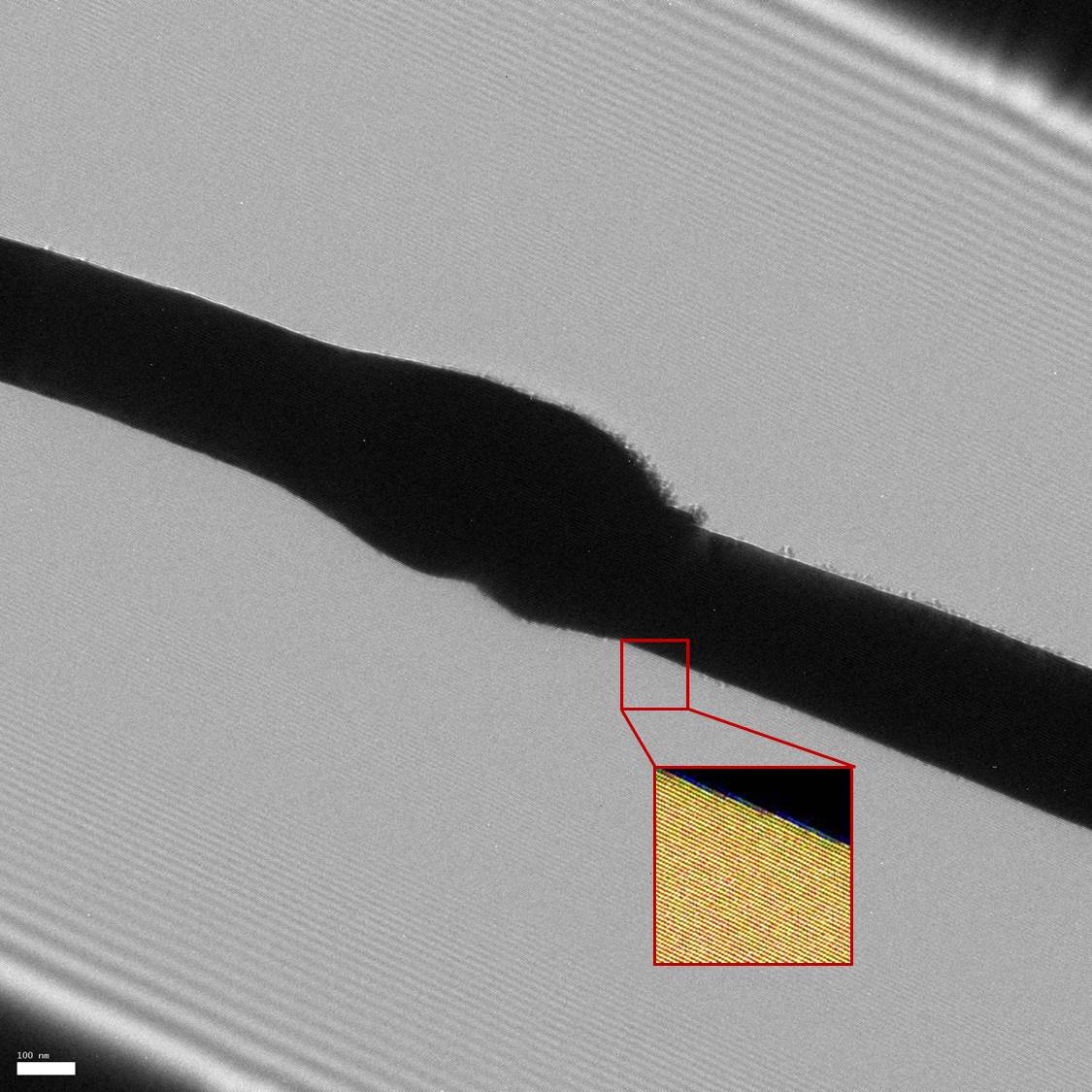


**Fig. S4** Representative off-axis electron hologram recorded from the Z-shaped device.


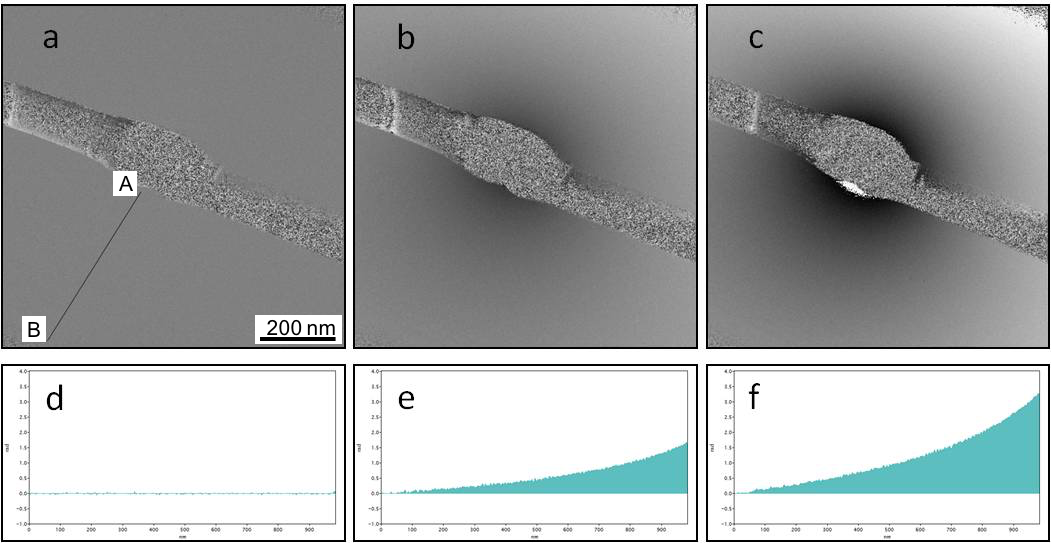


**Fig. S5** Reconstructed phase images recorded using off-axis electron holography for currents of a) 0, b) 2 and c) 4 mA. Noise and phase unwrapping errors are present within the boundary of the wire, where the amplitude is low. d) - f) Corresponding phase shift profiles measured between points A and B from images a) - c), respectively.


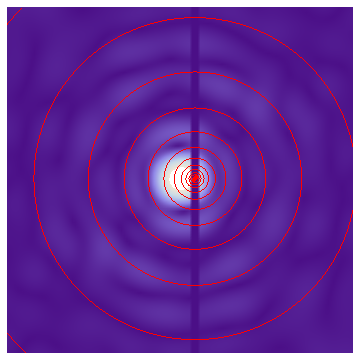


**Fig. S6** NCP power spectrum, with 0.25 rad phase contours superimposed, for a TAPP device operated at 1 mA. The lateral displacement and the size of the wire have been augmented for illustrative purposes.


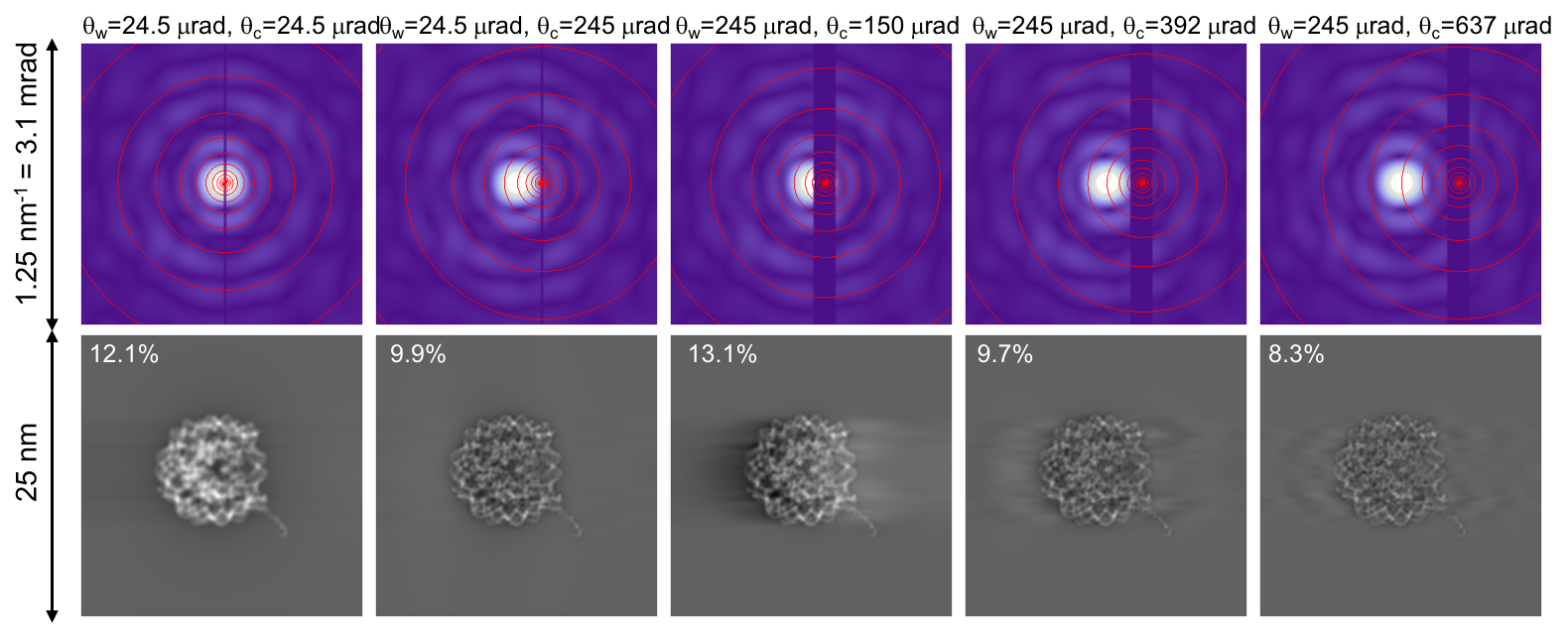


**Fig. S7** Top row: NCP power spectrum with 0.25 rad phase contours superimposed for a TAPP device operated at -0.6 mA, as the angular aperture of the wire and its lateral position are varied progressively. (The angles are reported at the top of the figure). Bottom row: corresponding image simulations, with contrast values indicated.
